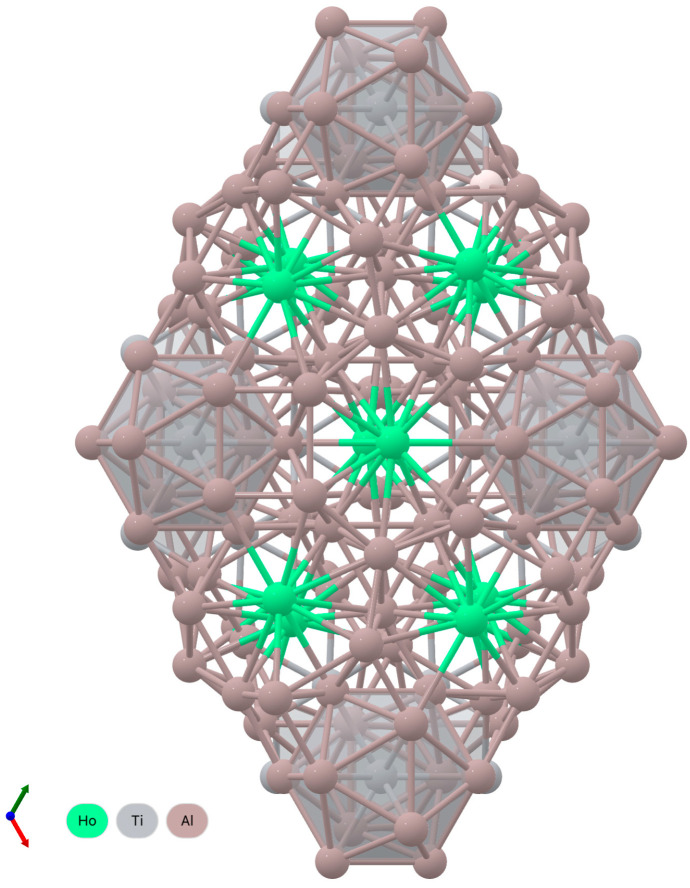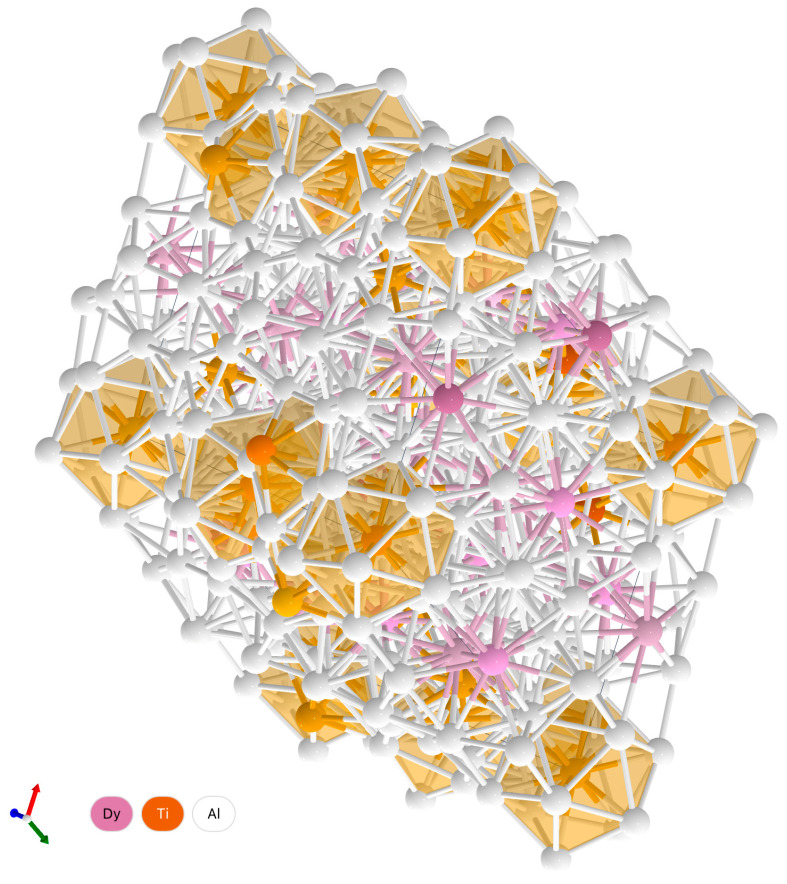# Influence of Dy and Ho on the Phase Composition of the Ti-Al System Obtained by ‘Hydride Technology’

**DOI:** 10.3390/ma15238584

**Published:** 2022-12-01

**Authors:** Natalia Karakchieva, Alina Artemenko, Sergei Sokolov, Ivan Amelichkin, Alexey Knyazev, Alexander Vorozhtsov, Yuri Abzaev, Victor Sachkov, Irina Kurzina

**Affiliations:** 1 Chemical Technology Laboratory, National Research Tomsk State University, 36 Lenin Avenue, 634050 Tomsk, Russia; 2 Laboratory of Metallurgy Nanotechnologies, National Research Tomsk State University, 36 Lenin Avenue, 634050 Tomsk, Russia; 3 Faculty of Chemistry, National Research Tomsk State University, 36 Lenin Avenue, 634050 Tomsk, Russia; 4 Material Research Centre for Collective Use, Tomsk State University of Architecture and Building, Solyanaya 2, 634003 Tomsk, Russia; 5 Smart Materials and Technologies Institute, National Research Tomsk State University, 36 Lenin Avenue, 634050 Tomsk, Russia

**Keywords:** hydride technology, intermetallides, Ti-Al, Ti-Al-Dy, Ti-Al-Ho, Dy_6_Ti_4_Al_43_, Ho_6_Ti_4_Al_43_

## Abstract

The manuscript describes the phase composition, microstructure, some physical and mechanical properties of the Ti-Al system with addition of 2 at. % Dy (TAD) and Ho (TAH) obtained by “hydride technology”. Phase diagrams for Ti-Al-Dy and Ti-Al-Ho at a temperature of 1150 °C and basic properties for ternary phases Dy₆Ti₄Al₄₃ and Ho₆Ti₄Al₄₃ were calculated. A crystallographic database of stable and quasistable structures of the known elemental composition was created in the USPEX-SIESTA software by means of an evolutionary code. The calculations show that adding REM leads to a significant stabilizing effect in each Ti-Al-Me (Me = Dy, Ho) system without exception. It has been established that the lattice energies of AlTi_3_Ho and AlTi_3_Dy are, respectively, equal to: EAl_4_Ti_12_Dy_3_ = −32,877.825 eV and EAl_4_Ti_12_Dy_3_ = −31,227.561 eV. In the synthesized Ti_49_Al_49_Ho_2_ compound, the main phases include Al-Ti, Al_3_Ti_3_ and Al_4_Ti_12_Ho_3_ and the contributions to the theoretical intensity are equal to 44.83, 44.43 and 5.55%, respectively. Ti_49_Al_49_Dy_2_ is dominated by the Al-Ti, Al_3_Ti_3_ and Al_4_Ti_12_Dy phases, whose contributions are equal to 65.04, 16.88 and 11.2%, respectively. The microhardness of TAD and TAN specimens is 1.61 ± 0.08 and 1.47 ± 0.07 GPa, respectively.

## 1. Introduction

Strict requirements imposed on the characteristics of the final material result in the necessity to develop new or improve existing alloys with increased physical and mechanical properties. This is associated with the widespread demand for such alloys in shipbuilding, mechanical engineering, aerospace, and other industries [1,2,3]. Titanium-aluminum (Ti-Al) alloys with additives of various rare earth metals (REM) are attractive materials meeting these requirements.

Obviously, the final properties of the material will depend on the chemical composition of the initial components. The search for a production technology depends on the set tasks, which include a high level of mechanical properties, manufacturability, resistance to corrosion and flammability. The increased operating temperatures of various products actualize the task of creating alloys with high strength and heat-resistant characteristics. Such properties can be achieved by adding various REM. REM with incomplete d-shells and having close atomic diameters with other components of the alloy allow forming complex-alloyed solid solutions and contribute to the formation of a large number of intermetallic compounds [4]. It should be noted that the addition of REM in alloys is an effective technique to reduce anisotropy. The study of the REM magnetic properties is of great interest from the point of view of understanding the fundamental physics of magnetic phenomena. Holmium (Ho) and dysprosium (Dy) have the maximum values of atomic magnetic moments in the lanthanide series [5]. The alloys of these REM are considered the basis of the best magnetostrictive materials.

Ho and its compounds are widely used in nuclear power engineering and industry. It is used for storage and transportation of liquefied gases, hydrogen, and its isotopes [6]. Magnetic cooling attracts considerable attention due to its high efficiency and environmental friendliness [7,8]. The addition of Ho can significantly reduce the alloy resistivity [9] and plays an important role in increasing its strength [10]. For instance, the addition of Ho to the Al-Cr-N system increases the oxidation resistance of coatings [11]. It can also increase the thermal stability of the alloy due to the formation of an amorphous and nanocrystalline phase [12]. In addition, it is worth noting that various ternary intermetallides are formed in the Al-Cr-Ho system. These intermetallics demonstrate promising electronic and magnetic properties [13]. The alloys based on REM show excellent magnetocaloric properties because of their complex electronic structure [14,15,16]. It is assumed that due to the low solubility of Ho in intermetallics, it will react with the melt during solidification and form phases rich in Ho [17]. This may contribute to enhancing the mechanical properties.

Dy, as well as Ho, is widely used in the production of magnets and in nuclear power engineering. It has a high degree of neutron absorption and a number of specific properties [18,19,20,21]. Dy is resistant to demagnetization at high temperatures. Iron-based alloys with addition of Dy are used as magnetostrictive materials. The alloys with Dy are a promising material for electronic and photonic devices [19,20,21,22,23,24,25,26]. It is also used for the safe liquefaction of natural gas using magnetic refrigeration [27,28]. A thermostable phase is formed in a solid aluminum solution with addition of Dy. This phase can suppress recrystallization, grain growth and significantly improve the mechanical properties of aluminum alloys at high temperatures [29,30].

The most widespread and effective alloying addition is scandium (Sc). Ho and Dy are promising and effective substitute for expensive scandium. Currently, ternary systems with the addition of these REM have been insufficiently studied. Only the crystal structure and magnetic properties of ternary compounds are mentioned. Therefore, the study of the REM influence on the structure formation and properties of titanium alloys is a relevant and promising direction in the development of modern materials science.

Hydride technology (HT)—a new way to obtain metallic materials. The advantage of the technology is possible to obtain compounds from elements whose melting points vary significantly. This is achieved using metal hydrides in the final stage.

In this way, the purpose of this study is a theoretical and experimental study of the process for obtaining the Ti-Al-REM ligature by “hydride technology”, analysis of the phase composition, microstructure, and some physical and mechanical properties of the Ti-Al system with addition of 2 at.% Ho and Dy.

## 2. Materials and Methods

### 2.1. Obtaining Alloy

This work investigates the structural state and quantitative phase analysis of the 49%Ti-49%Al-2%Dy (at.%, hereinafter referred to as TAD) and 49%Ti-49%Al-2%Ho (at %, hereinafter referred to as TAH) system, synthesized by the HT. The HT allows obtaining binary Ti-Al systems of a specified composition, as well as ternary Ti-Al-REM systems with a controlled content of REM.

To obtain Ti-Al-Me (Me = Dy, Ho) systems, powders of Ti (purity 99.2%), Dy (purity 99.9%) and Ho (purity 99.9%) were used as raw materials. Hydrides were obtained in a hydrogen stream (purity 99.9%) at 420 °C. Dy or Ho hydride was ground in an agate mortar before grinding. The obtained Ti, Dy, and Ho hydrides were re-hydrogenated for complete hydrogenation after grinding. Then, the resulting hydrides were thoroughly mixed with the Al powder (purity 99%). To obtain metal hydrides in a compact state, hydride powders were received using a laboratory extruder (LabTools, St. Petersburg, Russia, 2019) at a pressure of 4 × 10^6^ Pa. The specimens were placed in the reactor at a vacuum of 0.01 Pa and heated to 1150 °C.

### 2.2. Research Methods

X-ray diffraction analysis (XRD) of the obtained specimens (Ti-Al-REM) was performed using a DRON4–07 diffractometer with CuKα radiation in accordance with the Bragg–Brentano scheme in increments of 0.020 °C. The structural state and quantitative content of phases were identified by the Rietveld method using reflex [31]. The GSAS, PSW and XPOWDER were used as databases to determine phases. In the case of the Rietveld method, the maximum possible number of parameters was varied. The background radiation on the diffraction patterns was approximated by a polynomial of the 20th degree. The full-profile integral intensity of the reference phases was evaluated in a self-consistent manner. The reference lattices were selected from the crystallographic COD database [32].

Since there were no Ti-Al-REM compounds in COD database, the list of reference standards was supplemented with predicted structures obtained in the USPEX code with the SIESTA interface [33]. The lattices of the fixed Ti_49_Al_49_REM_2_ composition, used later for qualitative analysis of the phase content of synthesized Ti-Al-REM alloys, were predicted in the work. The stability of the reference lattices was assessed both in the USPEX-SIESTA software and in the CASTEP code [34]. The total energy of the lattices was determined at 0K. Calculations of the orbitals of the electronic states in CASTEP, the distribution of the one-electron density and the energy of the ground state were self-consistent. The valence electron wave functions of Ti-Al-REM atoms were analyzed in the plane wave cutoff parameters of kinetic energy, equal to 330 eV. The convergence in the total energy was ~0.5 × 10^−^^6^ eV/atom.

Computational modeling tools such as density functional theory (DFT) methods implemented in the Vienna Ab Initio Simulation Package (VASP) program (version VASP.6.X.) were used to calculate the phase diagrams. Having calculated the energies of known compounds in a certain chemical system, one can construct a phase diagram at a temperature T = 0 K and a pressure *p* = 0 atm. It should be noted that it is possible to draw approximate conclusions about the final phase diagrams at a given temperature and pressure for a system consisting mainly of solid phases that are free with respect to the gaseous element.

The microstructure of the specimens (Ti-Al-REM) was determined using a “JEM-2100F” (JEOL Ltd., Tokyo, Japan) microscope with energy-dispersion spectral (EDS) analysis. The results of morphology and elemental composition of the specimen surface were presented in the form of energy-dispersive X-ray spectra (EDX) using a QUANTA 200 3D (FEI Company, Hillsborough, OR, USA). The microhardness of the alloy specimens was determined using a PMT-3M microhardness tester (LOMO, JSC, St. Petersburg, Russia) at a load of 200 g by the Vickers method. Crystal structures Ho₆Ti₄Al₄₃ (mp-1212360) and Dy₆Ti₄Al₄₃ (mp-567159) were described using the open database Materials Project.

## 3. Results and Discussion

A crystallographic database of stable and quasistable structures of the known elemental composition was created in the USPEX-SIESTA software (version 10.5) by means of an evolutionary code. The reference standards from the COD database, as well as super cells (Al_4_Ti_12_Ho_3_ and Al_4_Ti_12_Dy_3_) found based on Ti_49_Al_49_REM_2_ composition, were used for the quantitative phase analysis (QFA) by the Rietveld method. QFA with embedded REM is complicated by the absence of crystallographic information in the COD database and literature that would allow identifying their concentration.

Table 1 and Table 2 show the reference and refined states of Al-Ti, Al_3_Ti_3_ and Ti_49_Al_49_REM_2_ (structural parameter, volume and lattice energy, space group, fraction and degree of reliability (R_wp_)). The reference and refined lattices are slightly different.

Lattice predictions in the USPEX-SIESTA software showed that the Al_3_Ti_3_ lattice with embedded REM in the interstices [0.5:0.5:0.5] could be attributed to quasistable structures based on Ti_49_Al_49_REM_2_ elemental composition. Additionally, quantum chemical calculations of the energy were carried out both in the initial state and by embedded REM in the CASTEP code. It has been established that the lattice energies are equal to: EAl_4_Ti_12_Dy_3_ = –32,877.825 eV and EAl_4_Ti_12_Dy_3_ = –31,227.561 eV. The calculations show that adding REM into these interstices is possible and leads to a significant stabilizing effect in each Ti-Al-REM system without exception. It is interesting to note that the increase in the binding energy is accompanied by a significant polarization of Millikan charges. Millikan charges on nanoadditives are equal to [(+0.48)Ho], [(+0.73)Dy], and on the atoms of the main elements they are [(−0.19)Al, (−0.06)Ti] and [(−0.17)Al, (−0.13)Ti] in ternary compounds Ti_49_Al_49_Ho_2_ and Ti_49_Al_49_Dy_2_, respectively. The charges are given in Coulomb units; the charge distribution is uniform on the atoms of the same grade. The charge polarizations in the mentioned systems is individual, both negative and positive Millikan charges can be concentrated on the REM atoms.

The Al-Ti crystal lattice has a P4/MMM (Tetragonal) space group, Al_3_Ti_3_ crystal lattice has a P1 (Triclinic) space group, and Ti_49_Al_49_REM_2_ has a P6/MMM (Hexagonal) space group.

**Figure 1:**
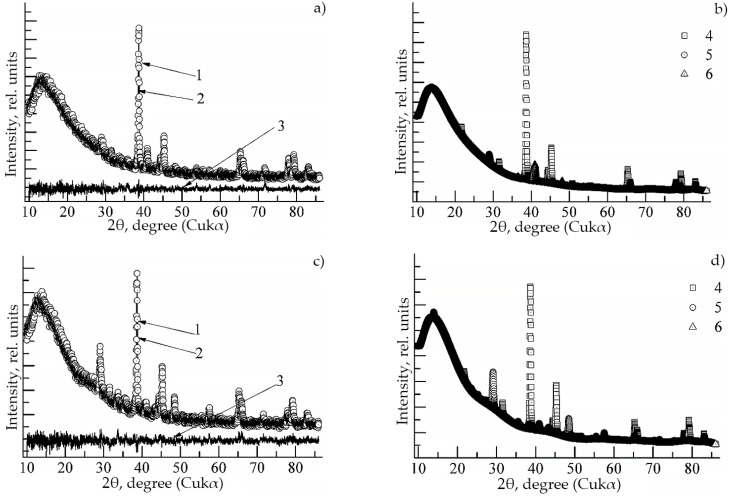
Diffraction patterns of alloys: TAD (**a**,**b**); TAH (**c**,**d**). The figures indicate: 1—experiment, 2—total model intensity, and 3—difference between the intensities. Numbers 4, 5 and 6 are contributions to the integral intensity of single phases.

**Figure 2:**
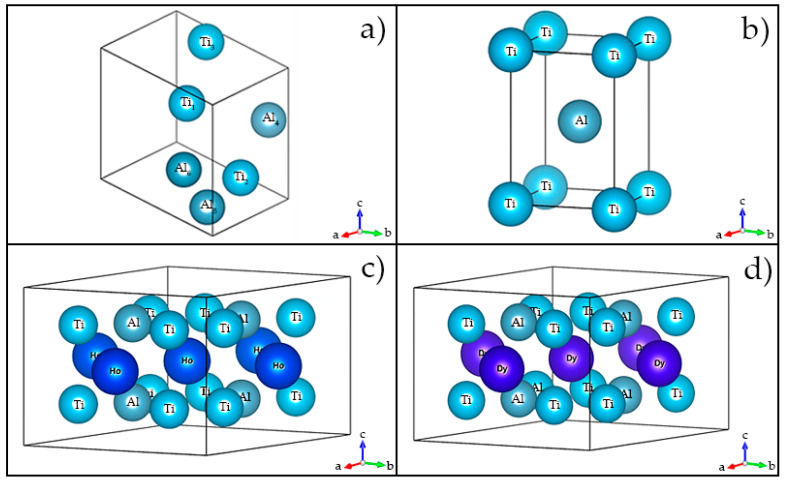
The 3D reference lattices of the Al-Ti alloys with addition of REM: Ho (**a**) Al_3_Ti_3_, (**b**) Al-Ti, (**c**) Al_4_Ti_12_Ho_3_, and (**d**) Al_4_Ti_12_Dy_3_.

The QFA of contributions to the integral intensity of individual phases (Table 1 and Table 2 and Figure 1) showed that with a high degree of reliability (R_wp_ < 7.2%), the experimental diffractograms of synthesized compounds Ti_49_Al_49_Ho_2_ and Ti_49_Al_49_Dy_2_ are well approximated by the integral (theoretical) intensity. This is also evidenced by the intensity differences (Figure 1 and Diagrams 3). However, the contributions of individual phases differ in different systems. Figure 1b,d also show the calculated intensities of single phases. In the synthesized Ti_49_Al_49_Ho_2_ compound, the main phases include Al-Ti, Al_3_Ti_3_ and Al_4_Ti_12_Ho_3_. The contributions to the theoretical intensity are equal to 44.83, 44.43 and 5.55%, respectively (Table 1). Ti_49_Al_49_Dy_2_ is dominated by the Al-Ti, Al_3_Ti_3_ and Al_4_Ti_12_Dy phases, whose contributions are equal to 65.04, 16.88 and 11.2% (Table 2), respectively. The 3D lattices with the spatial distribution of atoms are shown in Figure 2.

The obtained results indicate that the synthesized Ti_49_Al_49_Ho_2_ and Ti_49_Al_49_Dy_2_ compounds are dominated by binary AlTi-based compounds, whose proportion exceeds 0.90 of the total content. The parameters of the refined lattices and the volume of Al-Ti and Al_3_Ti_3_ differ slightly from the values in the reference state. In the Al_4_Ti_12_Ho_3_ alloy, the lattice volume increased mainly due to the growth of the *z* coordinate. Complete structural information is known for all the reference and predicted lattices.

It has been established that Ti_49_Al_49_Ho_2_ and Ti_49_Al_49_Dy_2_ compounds are formed during the synthesis of Ti-Al-REM systems with a controlled content of REM (Ho_2_, Dy_2_). It was found that phases based on Al-Ti predominate in the studied specimens. The total proportion of phases is 0.89 and 0.76. The ternary Ti_49_Al_49_Ho_2_ and Ti_49_Al_49_Dy_2_ alloys have a lower content. The total proportion of which was 0.05 and 0.16. The existence and quasistability of ternary systems are predicted in the USPEX-SIESTA and CASTEP codes.

**Figure 3:**
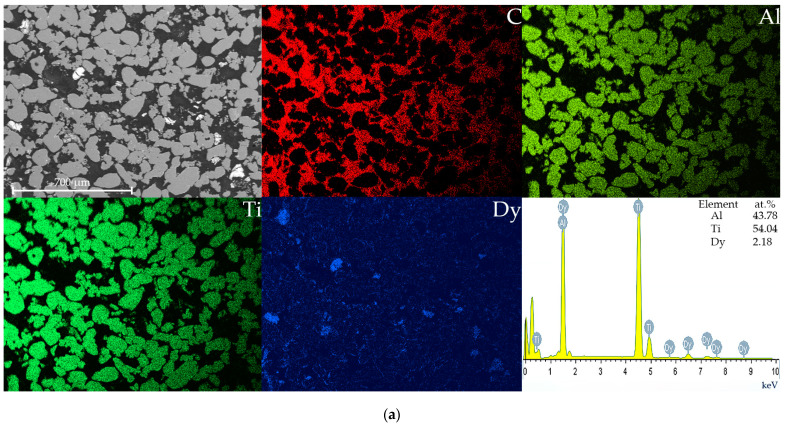

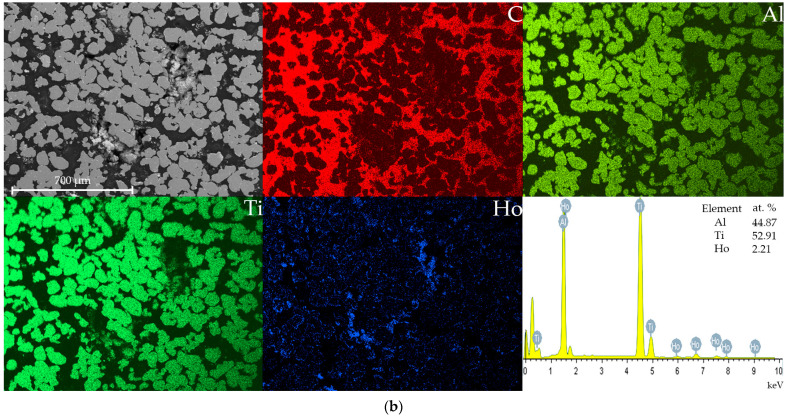
Transmission electron microscopy (TEM) patterns of the TAD (**a**) and TAH (**b**) alloys with super-spectral surface and with EDS analysis spectrum of relevant areas.

The microstructure of the TAD and TAH alloys with characteristic X-ray radiation spectra is shown in Figure 3. The composite materials contain pores. A color gradient is observed in the SEM patterns. Gray areas correspond to the alloy and dark areas correspond to the porous space.

In the material, there are unevenly distributed areas with a predominance of Dy (luminous areas in black-white image) up to 300 μm in size. The consequence of the modifying effect of Dy is a relatively larger average grain size (150 µm) of the studied experimental Dy-containing gamma alloys in contrast to alloys with Sc and Y microadditives (100 µm) [35,36]. Grains with an average size of 110–130 µm were formed on the TAH specimen surface. The addition of Ho to the Ti-Al system leads to the formation of smaller grains compared to Dy. Rounded Dy-containing phases are observed in different parts of the matrix during EDS analysis directly in the places of accumulation of various kinds of inclusions (Figure 3). It was established that the TAD alloy matrix consists only of Al and Ti particles during local EDS analysis. The results obtained are in good agreement with the data [37]. Dy and Ho are concentrated at grain boundaries as shown in Figure 3.

It should be noted that the presence of carbon is due to the use of a conductive compound into which the specimen is pressed. Since the specimens have a porous structure, the compound fills these pores during processing (grinding-polishing) and is identified as carbon during mapping.

**Figure 4:**
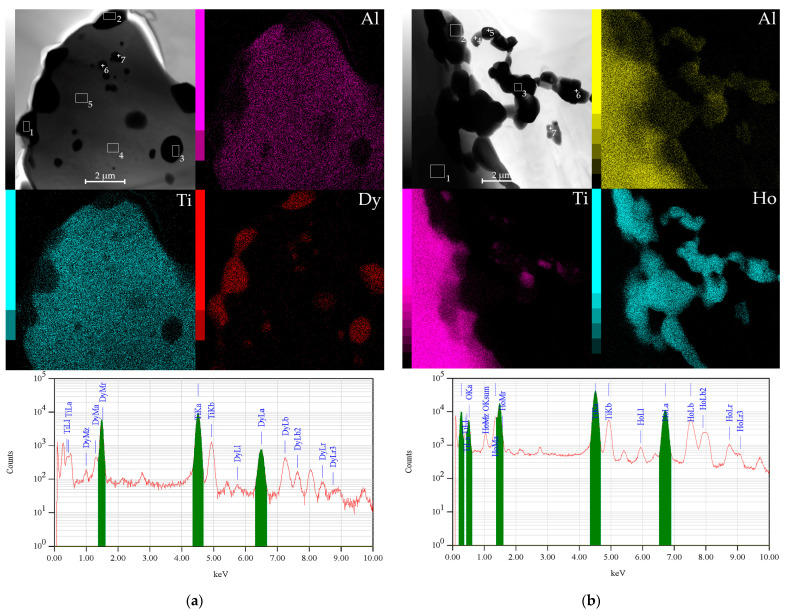
Transmission electron microscopy (TEM) images with super-spectral surface and with energy-dispersive spectral analysis (EDS) spectrum of relevant areas: the TAD alloy (**a**); the TAH (**b**) alloys.

The particles morphology formed at grains boundary has been studied in detail. Figure 4 shows that dark particles with an average size of 0.51 µm are located on the surface, and larger particles (approximately 1.14 µm) are located at the grain boundary for the TAD specimen. For the TAH specimen, the average size of the formed particles are 1–1.3 µm. Moreover, the particles enriched with Ho (in the TAH specimen) form clusters (chains) among themselves.

The EDS results for the TAD and TAH specimens are shown in Table 3. The elemental analysis showed that the grains of the TAD system differed a little in chemical composition. It is shown that the grains richest by Dy are located at the boundary (Spectrum 1–3). According to the elemental analysis of local areas in the TAH specimens, individual dark particles located in a chain are compounds of Dy, Ti and Al. Individual particles formed sequentially are Ho particles enriched in Ti and Al. The brighter particles are enriched with oxygen and are compounds of the formed oxides.

**Figure 5:**
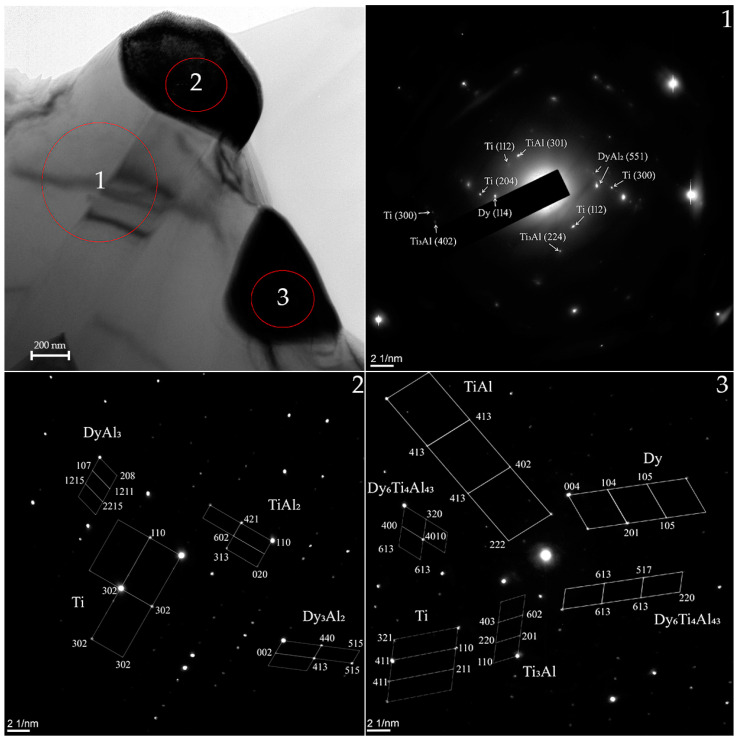
TEM patterns of the TAD alloy, selected area electron diffraction (SAED) patterns of the TAD alloy in the relevant region (1)–(3).

On Figure 5 shows a bright-field pattern obtained in the volume of a TAD specimen. The interpretation of the microdiffractogram was carried out for more detailed identification (Figure 5, regions 1–3). It can be seen that a lamellar structure begins to form in the TAD pattern, which is not clearly represented. The phases Ti (300), Ti (112), Ti_3_Al (402), Ti_3_Al (224), TiAl (301), DyAl_2_ (551) and Dy (114) were found during the surface interpretation. According to the interpretation of the obtained data in the spectral region (2), the TiAl_2_ (Cmmm, orthorhombic), Dy_3_Al_2_ (P42nm, tetragonal), and Dy_3_Al_2_ (3m, rhombohedral) phases were identified. The following phases were found in spectral region (3): Ti-Al (P4/mmm, tetragonal), Ti_3_Al (P63/mmc, hexagonal), Ti (Im-3m, cubic), Dy (P63/mmc, hexagonal), and also Dy_6_Ti_4_Al_43_ (P63/mmc, hexagonal).

**Figure 6:**
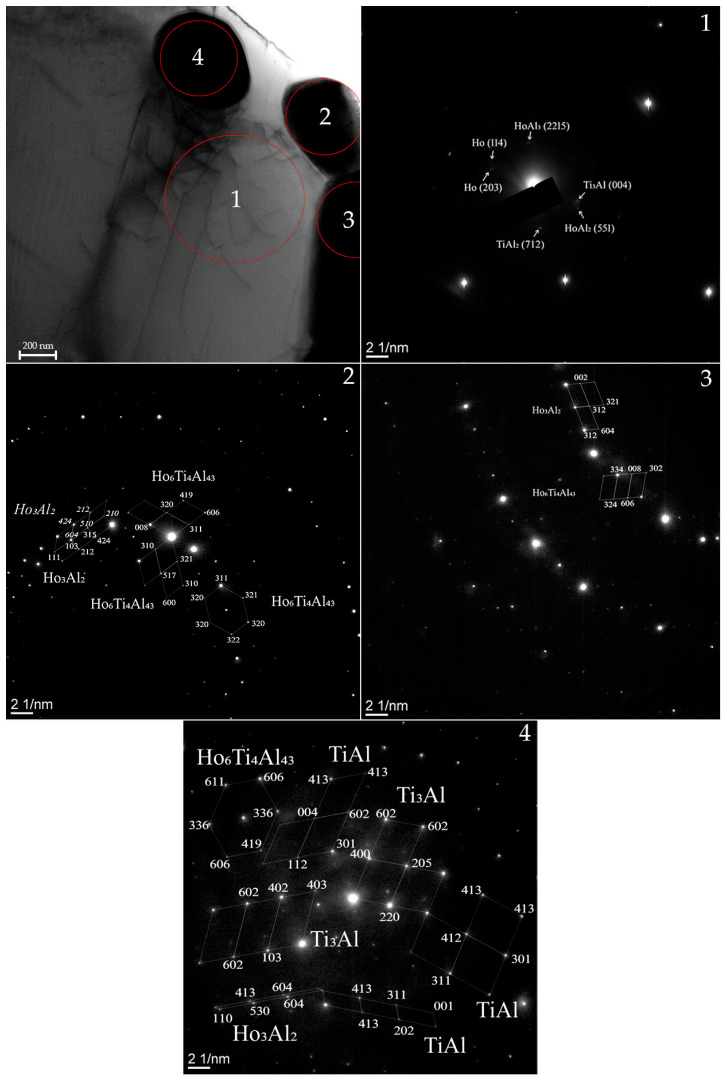
TEM patterns of the TAD alloy, selected area electron diffraction (SAED) patterns of the TAD alloy in the relevant region (1)–(4).

A detailed analysis of the TAH specimen showed the presence of dislocations on the surface (Figure 6). In this way, phases TiAl_2_ (712), Ti_3_Al (004), Ho (114) Ho (203), HoAl_3_ (2215), HoAl_2_ (551) were identified in the surface composition of the spectral region (1). The authors think that the formation of dislocations is associated with the product crystallization. In the process of which the formation of dispersed particles and lamellar structures occurs that causes stress and increased defectiveness of the alloy. Analysis in spectral region (2) showed the presence of the Ho_3_Al_2_ (P42nm, tetragonal) and Ho_6_Ti_4_Al_43_ (P63/mmc, hexagonal) phases. The similar composition of the phases is typical for the spectral region (3). These particles are located close to the grain edge and are mainly represented by phases consisting of Ho and Al. The particle in the volume differs in phase composition. In addition to Ho_3_Al_2_ (P42nm, tetragonal) and Ho_6_Ti_4_Al_43_ (P63/mmc, hexagonal), the presence of Ti-Al (P4/mmm, tetragonal) and Ti_3_Al (P63/mmc, hexagonal) was detected during identification of TEM results.

**Figure 7:**
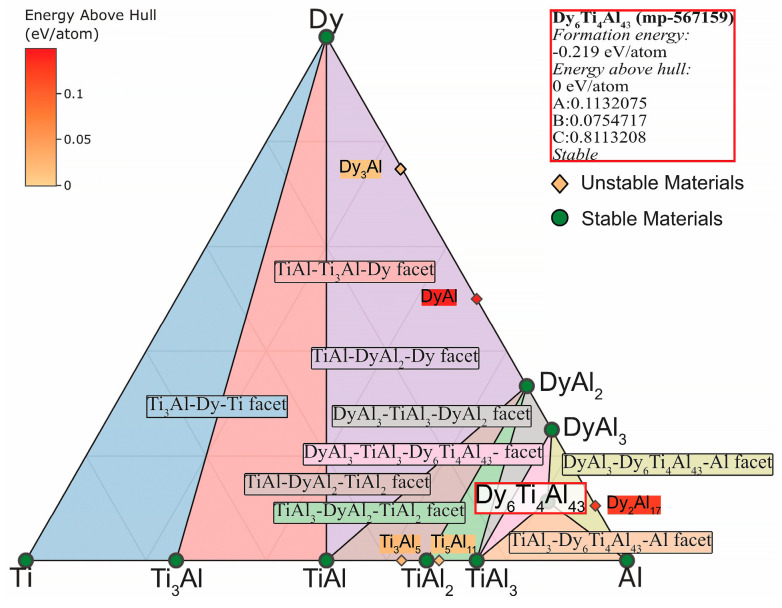
Ti-Al-Dy phase diagram at a temperature of 1150 °C.

**Figure 8:**
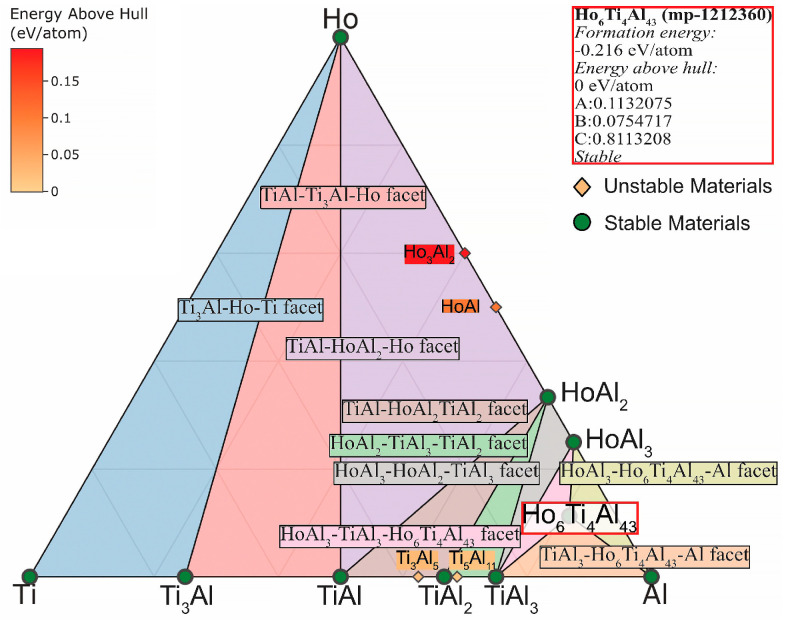
Ti-Al-Ho phase diagram at a temperature of 1150 °C.

The phase diagrams were calculated and plotted at a temperature of 1150 °C (temperature of vacuum annealing of the specimens) in the case of ternary TAD and TAN systems. This was done to determine the compounds formation area of isotypes Dy_6_Ti_4_Al_43_ and Ho_6_Ti_4_Al_43_. Figure 7 shows that the Dy_6_Ti_4_Al_43_ compound is stable. In addition, the formation of stable phases DyAl_3_ and DyAl_2_ in this area is theoretically feasible. Moreover, the Dy_6_Ti_4_Al_43_ compound has common faces: DyAl_3_-Dy_6_Ti_4_Al_43_-Al, TiAl_3_-Dy_6_Ti_4_Al_43_-Al and DyAl_3_-TiAl_3_-Dy_6_Ti_4_Al_43_. Similarly, it is theoretically plausible to formation the stable phases HoAl_3_ and HoAl_2_ with common faces: HoAl_3_-Ho_6_Ti_4_Al_43_-Al, TiAl_3_- Ho_6_Ti_4_Al_43_-Al and HoAl_3_-TiAl_3_-Ho₆Ti₄Al₄₃ in the TAH specimen (Figure 8). The obtained diagrams are consistent with the data for the TAD and TAH systems [38,39,40,41].

Theoretical calculations were carried out and the main properties of the Ho_6_Ti_4_Al_43_ and Dy_6_Ti_4_Al_43_ compounds were described (Table 4). The Ho_6_Ti_4_Al_43_ and Dy_6_Ti_4_Al_43_ phases crystalize in the hexagonal P6₃/mcm space group (No. 193). They have almost equal formation energy of ≈−0.340 eV/atom and are non-magnetic materials. The total magnetization is 0.33 and 0.41 µB/f.u., respectively. They are thermodynamically stable. The calculated density is 4.12 (Ho_6_Ti_4_Al_43_) and 4.08 g·cm⁻^3^ (Dy_6_Ti_4_Al_43_).

Such compounds belong to isotype compounds of the Ho_6_Mo_4_Al_43_ (P63/mcm, hexagonal) type. There are data on the magnetic properties of Dy_6_Ti_4_Al_43_ and Ho_6_Ti_4_Al_43_ systems [44]. According to [45], the Dy_6_Ti_4_Al_43_ compound has paramagnetic properties and Ho_6_Ti_4_Al_43_ ferro- or paramagnetic properties. The calculations presented in Table 4 show that the Dy_6_Ti_4_Al_43_ and Ho_6_Ti_4_Al_43_ structures are stable. They do not have magnetic ordering, but the total magnetization is 0.33 and 0.41 µB/f.u, respectively.

The microhardness was measured for TAD and TAN specimens. It has been established that the microhardness for TAD is 1.61 ± 0.08 GPa, and for TAH is 1.47 ± 0.07 GPa. The microhardness for the Ti-Al system obtained by HT is 1.23 ± 0.06 GPa [35]. The increased microhardness may be associated with a change in the phase composition [41].

## 4. Conclusions

In this work, materials from the Ti-Al-Me (Me = Dy, Ho) system were obtained by “hydride technology”. The phase composition, microstructure, some physical and mechanical properties of the Ti-Al system with addition of 2 at % Ho or Dy were studied.

1. The addition of 2 at.% Dy to Ti-Al system leads to the formation of the following phases: Ti (300), Ti (112), Ti_3_Al (402), Ti_3_Al (224), TiAl (301), DyAl_2_ (551), Dy (114), TiAl_2_ (Cmmm, orthorhombic), Dy_3_Al_2_ (P42nm, tetragonal), Dy_3_Al_2_ (3m, rhombohedral), Ti-Al (P4/mmm, tetragonal), Ti_3_Al (P63/mmc, hexagonal), Ti (Im-3m, cubic), Dy (P63/mmc, hexagonal), as well as Dy_6_Ti_4_Al_43_ (P63/mmc, hexagonal).
2. The addition of 2 at % Ho to Ti-Al system leads to the formation of the following phases: TiAl_2_ (712), Ti_3_Al (004), Ho (114) Ho (203), HoAl_3_ (2215), HoAl_2_ (551), Ho_3_Al_2_ (P42nm, tetragonal), Ho_6_Ti_4_Al_43_ (P63/mmc, hexagonal), TiAl (P4/mmm, tetragonal) and Ti_3_Al (P63/mmc, hexagonal).
3. A crystallographic database of stable and quasistable structures of the known elemental composition was created in the USPEX-SIESTA software by means of an evolutionary code. The calculations show that adding REM leads to a significant stabilizing effect in each Ti-Al-REM system without exception. It has been established that the lattice energies are equal to: EAl_4_Ti_12_Dy_3_ = −32,877.825 eV and EAl_4_Ti_12_Dy_3_ = −31,227.561 eV.
4. The differences and commonality in the three-component TAD and TAH phase diagrams calculated using the Materials Project open database at a temperature of 1150 °C have been considered. The presence of ternary phases Dy₆Ti₄Al₄₃ and Ho₆Ti₄Al₄₃ with common faces MeAl_3_-Me₆Ti₄Al₄₃-Al, TiAl_3_-Me₆Ti₄Al₄₃-Al and MeAl_3_-TiAl_3_-Dy₆Ti₄Al₄₃ (Me = Dy, Ho) has been shown.
5. The properties for ternary Dy₆Ti₄Al₄₃ and Ho₆Ti₄Al₄₃ phases have been theoretically calculated. The Ho₆Ti₄Al₄₃ phase has large values of predicted formation energy, Total magnetization, volume, bond length. However, the density of such phase is less.
6. The addition of 2 at.% Dy increases the value of microhardness (1.61 ± 0.08 GPa) as compared with the case of adding 2 at.% Ho (1.47 ± 0.07 GPa).

## Figures and Tables

**Table 1:** Structural parameters of the lattices in the TAH alloy.

Phase	State	a, Å	b, Å	c, Å	α	β	γ	V, Å^3^	E, eV	Space Group	Fraction, %	R_wp_, %
Al-Ti	Reference	2.837	2.837	4.059	90.00	90.00	90.00	32.677	−1660.341	P4/mmm, Tetragonal	44.83	6.302
	Refined	2.826	2.826	4.072	90.00	90.00	90.00	32.525				
Al_3_Ti_3_	Reference	6.294	4.109	4.292	113.31	92.71	92.50	101.596	−4954.206	P1, Triclinic	44.34	
	Refined	6.267	4.093	4.288	113.70	91.93	92.44	100.44				
Al_4_Ti_12_Ho_3_	Reference	5.764	5.764	4.664	90.00	90.00	120.00	134.20	−32,877.825	P6/mmm, Hexagonal	5.55	
	Refined	5.731	5.731	4.773	90.00	90.00	120.00	135.77				

**Table 2:** Structural parameters of the lattices in the TAD alloy.

Phase	State	a, Å	b, Å	c, Å	α	β	γ	V, Å^3^	E, eV	Space Group	Fraction, %	R_wp_, %
Al-Ti	Reference	2.837	2.837	4.059	90.00	90.00	90.00	32.677	−1660.341	P4/mmm, Tetragonal	65.04	6.504
	Refined	2.826	2.826	4.074	90.00	90.00	90.00	32.537				
Al_3_Ti_3_	Reference	6.339	4.150	4.234	113.36	93.36	92.52	101.79	−4978.606	P1, Triclinic	16.88	
Al_4_Ti_12_Dy_3_	Reference	5.764	5.764	4.664	90.00	90.00	120.00	132.56	−31,227.561	P6/mmm, Hexagonal	11.20	
	Refined	5.771	5.771	4.657	90.00	90.00	120.00	134.34				

**Table 3:** Summary table of the EDS results.

TAD				TAH				
Spectrum	Element, at %			Spectrum	Element, at %			
	Ti	Al	Dy		Ti	Al	Ho	O
1	13.11	1.87	85.02	1	72.63	26.63	0.2	0.53
2	12.66	3.3	84.04	2	10.39	4.56	47.85	37.20
3	13.28	4.02	82.71	3	12.77	4.45	60.45	22.33
4	59.12	40.82	0.06	4	2.38	5.37	31.52	60.73
5	62.22	37.69	0.09	5	7.59	3.39	48.11	40.92
6	56.87	37.05	6.08	6	20.41	5.36	47.99	26.24
7	51.9	28.81	19.28	7	8.15	2.81	35.21	53.82

**Table 4:** Theoretically calculated properties of Ho₆Ti₄Al₄₃ and Dy₆Ti₄Al₄₃.

Properties	Ho₆Ti₄Al₄₃ [42]					Dy₆Ti₄Al₄₃ [43]				
Space Group	P63/mcm (No. 193)					P63/mcm (No. 193)				
Predicted Formation Energy	−0.338 eV/atom					−0.340 eV/atom				
Magnetic Ordering	Non-magnetic					Non-magnetic				
Total Magnetization	0.33 µB/f.u.					0.41 µB/f.u				
Thermodynamic Stability	Yes					Yes				
Density	4.12 g·cm⁻^3^					4.08 g·cm⁻^3^				
Volume	1887.56 Å^3^					1895.57 Å^3^				
Bond length	Ho–Ho—3.49 Å					Dy–Dy—3.51 Å				
	Ho–Ti—3.50 Å					Dy–Ti—3.52 Å				
	Ho–Al—from 3.08 to 3.47 Å					Dy–Al—from 3.08 to 3.49 Å				
Atomic Positions	Wyckoff	Element	X	Y	Z	Wyckoff	Element	X	Y	Z
	2b	Ti	0	0	0	2b	Ti	0	0	1/2
	6g	Ti	0	0.27	1/4	6g	Ti	0.73	0.73	3/4
	6g	Al	0	0.85	1/4	6g	Al	0	0.15	1/4
	8h	Al	2/3	1/3	0.13	8h	Al	1/3	2/3	0.63
	12i	Al	0.49	0.25	0	12i	Al	0.75	0.5	0
	12j	Al	0.6	0.45	1/4	12j	Al	0.4	0.85	3/4
	12k	Ho	0	0.47	0.1	12k	Dy	0.53	0	0.1
	12k	Al	0	0.84	0.62	12k	Al	0	0.75	0.97
	12k	Al	0	0.75	0.03	12k	Al	0.16	0	0.62
	24l	Al	0.39	0.24	0.16	24l	Al	0.84	0.61	0.84
Vizualization